# Water Extractable Phytochemicals from Peppers (*Capsicum* spp.) Inhibit Acetylcholinesterase and Butyrylcholinesterase Activities and Prooxidants Induced Lipid Peroxidation in Rat Brain *In Vitro*

**DOI:** 10.1155/2014/605618

**Published:** 2014-12-04

**Authors:** Omodesola O. Ogunruku, Ganiyu Oboh, Ayokunle O. Ademosun

**Affiliations:** ^1^Department of Biochemistry, Obafemi Awolowo University, Ile Ife 220282, Nigeria; ^2^Functional Foods and Nutraceuticals Unit, Department of Biochemistry, Federal University of Technology, PMB 704, Akure, Ondo State 340001, Nigeria

## Abstract

*Background*. This study sought to investigate antioxidant capacity of aqueous extracts of two pepper varieties (*Capsicum annuum* var. *accuminatum* (SM) and *Capsicum chinense* (RO)) and their inhibitory effect on acetylcholinesterase and butyrylcholinesterase activities. *Methods*. The antioxidant capacity of the peppers was evaluated by the 2,2′-azino-bis(3-ethylbenzthiazoline-6-sulphonic acid) (ABTS) radical scavenging ability and ferric reducing antioxidant property. The inhibition of prooxidant induced lipid peroxidation and cholinesterase activities in rat brain homogenates was also evaluated. *Results*. There was no significant difference (*P* > 0.05) in the total phenol contents of the unripe and ripe *Capsicum* spp. extracts. Ripe and unripe SM samples had significantly higher (*P* < 0.05) ABTS^*^ scavenging ability than RO samples, while the ripe fruits had significantly higher (*P* < 0.05) ferric reducing properties in the varieties. Furthermore, the extracts inhibited Fe^2+^ and quinolinic acid induced lipid peroxidation in rats brain homogenates in a dose-dependent manner. Ripe and unripe samples from SM had significantly higher AChE inhibitory abilities than RO samples, while there was no significant difference in the BuChE inhibitory abilities of the pepper samples. *Conclusion*. The antioxidant and anticholinesterase properties of *Capsicum* spp. may be a possible dietary means by which oxidative stress and symptomatic cognitive decline associated with neurodegenerative conditions could be alleviated.

## 1. Introduction

Alzheimer's disease (AD) is characterized by progressive cognitive, functional, and behavioral impairment, which evolves into a dramatic loss of most cortical and subcortical functions and, ultimately, death [1]. Demographic changes and the rise in life expectancy have resulted in the growth recorded in the number of individuals affected by AD. It is forecast that the worldwide number of elderly people suffering from dementia will rise to 63 million in 2030 and to 114 million in 2050 [2]. The socioeconomic impact associated with the global AD burden that cannot be estimated raises the need to find preventive and palliative means of treatment for sufferers.

Deficiency of the neurotransmitter acetylcholine at the synaptic cleft coupled with cholinergic decline in the hippocampal and cortical regions of the brain is associated with cognitive decline observed in AD [3], a situation which is further aggravated by deposition of amyloid proteins observed in senile plaques in the extracellular matrix of the brain [4]. There is evidence that cholinesterase inhibitors (ChEIs) may slow disease progression and hippocampal atrophy and may have disease-modifying effects [5]. Furthermore, treatments with ChEIs have been found to have a positive correlation with improved cognitive abilities with modest and significant therapeutic effects [6]. Thus, restoring the level of acetylcholine through inhibition of both major forms of cholinesterases, acetylcholinesterase (AChE), and butyrylcholinesterase (BuChE) is considered to be a useful therapeutic approach [7, 8]. While all approved ChEIs (donepezil, rivastigmine, and galantamine) inhibit both forms of cholinesterases, however, continued search among natural products has intensified due to adverse side effects associated with these drugs.

Furthermore, the mammalian brain is particularly sensitive towards oxidative damage, a result of the high oxygen demand and the high content of unsaturated lipids in the central nervous system [9] coupled with the reduced access to the antioxidant defense system and the high amount of redox-active transition metal ions [10, 11]. Oxidative stress in the central nervous system is an underlying cause of neurodegeneration and neural dysfunction [12]. This has resulted in considerable research efforts being put towards reducing the risks and event of oxidative stress with the use of antioxidants.

Peppers (*Capsicum *spp.), a genus of related plants, belong to the family Solanaceae and have a worldwide distribution. They are important as vegetable foods and spice and are considered an important source of nutrients in the human diet which are grown in tropical, subtropical, and temperate regions [13–15]. Various species and varieties occurring within the* Capsicum* genus depending on their shape, size, flavor, and hotness include* Capsicum annum*,* Capsicum frutescence*, and* Capsicum chinense*. Previous studies [16, 17] have highlighted some of the health benefits associated with the consumption of* Capsicum* spp. Therefore, this study sought to assess the ability of pepper fruits to interact with some enzymes (acetylcholinesterase and butyrylcholinesterase) which are implicated in AD pathology* vis-à-vis* the antioxidant capacity of the pepper fruits.

## 2. Materials and Methods

### 2.1. Materials

#### 2.1.1. Sample Collection

Fresh samples of two samples of peppers both ripe and unripe “sombo” (*Capsicum annuum *var.* accuminatum*) and “ata rodo” (*Capsicum chinense*) were collected from farm settlements around Akure Metropolis, Nigeria. Authentication of the samples was carried out at the Department of Biology, Federal University of Technology, Akure, Nigeria.

#### 2.1.2. Sample Preparation

The samples were washed and homogenized in distilled water (1 : 20 w/v). These were later centrifuged at 4000 rpm for five minutes, and the supernatant was collected which was stored at 4°C until further analysis.

#### 2.1.3. Chemicals and Reagents

Chemicals and reagents used such as thiobarbituric acid (TBA), 1,10-phenanthroline, deoxyribose, gallic acid, and Folin-Ciocalteau's reagent were procured from Sigma-Aldrich, Inc. (St Louis, MO), trichloroacetic acid (TCA) was sourced from Sigma-Aldrich, Chemie GmbH (Steinheim, Germany), dinitrophenylhydrazine (DNPH) was sourced from ACROS Organics (New Jersey, USA), and hydrogen peroxide, methanol, acetic acid, thiourea, CuSO_4_·5H_2_O, H_2_SO_4_, HCl, sodium carbonate, AlCl_3_, potassium acetate, Tris-HCl buffer, sodium dodecyl sulphate, FeSO_4_, potassium ferricyanide, and ferric chloride were sourced from BDH Chemicals Ltd. (Poole, England), while the water was glass distilled.

#### 2.1.4. Experimental Animals

Approval was obtained from the relevant departmental ethics committee responsible for the use of laboratory animals. The handling and the use of the animals were in accordance with NIH Guide for the care and use of laboratory animals. Adult male Wistar strain albino rats were purchased from the Department of Biochemistry, University of Ilorin. All animals were given ad libitum access to commercial diet and maintained on a 12 h light/dark cycle. All ethical standards regarding the use of animals in experimental procedures were strictly adhered to.

### 2.2. Methods

#### 2.2.1. Determination of Total Phenol Content

The total phenol content was determined according to the method of Singleton et al. [18]. Briefly, appropriate dilutions of the aqueous extracts were oxidized with 2.5 mL 10% Folin-Ciocalteau's reagent (v/v) and neutralized by 2.0 mL of 7.5% sodium carbonate. The reaction mixture was incubated for 40 min at 45°C and the absorbance was measured at 765 nm in the JENWAY UV-visible spectrophotometer. The total phenol content was subsequently calculated as gallic acid equivalent.

#### 2.2.2. *In Vitro* Antioxidant Studies

*2,2* ′ *-Azino-bis(3-ethylbenzthiazoline-6-sulphonic acid) (ABTS) Radical Scavenging Ability.* The ABTS^*^ scavenging ability of the extracts was determined according to the method described by Re et al. [19]. The ABTS^*^ was generated by reacting an (7 mmol/L) ABTS aqueous solution with K_2_S_2_O_8_ (2.45 mmol/L, final concentration) in the dark for 16 h and adjusting the absorbance at 734 nm to 0.700 with ethanol. 0.2 mL of appropriate dilution of the extract was added to 2.0 mL ABTS^*^ solution and the absorbance was measured at 734 nm after 15 min in the JENWAY UV-visible spectrophotometer. The Trolox equivalent antioxidant capacity (TEAC) was subsequently calculated.

#### 2.2.3. Determination of Reducing Property

The reducing property of the extracts was determined by assessing the ability of the extract to reduce FeCl_3_ solution as described by Oyaizu [20]. 2.5 mL aliquot was mixed with 2.5 mL 200 mM sodium phosphate buffer (pH 6.6) and 2.5 mL 1% potassium ferricyanide. The mixture was incubated at 50°C for 20 min, and then 2.5 mL 10% trichloroacetic acid was added. This mixture was centrifuged at 650 rpm for 10 min. 5 mL of the supernatant was mixed with an equal volume of water and 1 mL 0.1% ferric chloride. The absorbance was measured at 700 nm in the JENWAY UV-visible spectrophotometer. The ferric reducing antioxidant property was subsequently calculated as ascorbic acid equivalent.

#### 2.2.4. Lipid Peroxidation Assay

*Preparation of Tissue Homogenates.* The rats were decapitated under mild diethyl ether anaesthetic conditions and the whole brain tissue was quickly isolated and placed on ice and weighed. The tissue was subsequently homogenized in cold saline (1/10 w/v) with about 10 up-and-down strokes at approximately 1200 rev/min in a Teflon glass homogenizer. The homogenates were centrifuged for 10 min at 3000 rpm to yield pellets that were discarded, and a low-speed supernatant (S1) fraction was kept for lipid peroxidation assay [21].

#### 2.2.5. Lipid Peroxidation and Thiobarbituric Acid Reactions

The lipid peroxidation assay was carried out using the modified method of Ohkawa et al. [22]. Briefly 100 *μ*L S1 fraction was mixed with a reaction mixture containing 30 *μ*L of 0.1 M pH 7.4 Tris-HCl buffer extract (0–100 *μ*L) and 30 *μ*L of 250 *μ*M freshly prepared FeSO_4_ (the procedure was also carried out using 5 mM sodium nitroprusside and 15 mM quinolinic acid). The volume was made up to 300 *μ*L by water before incubation at 37°C for 1 h. The colour reaction was developed by adding 300 *μ*L 8.1% sodium dodecyl sulphate (SDS) to the reaction mixture containing S1, and this was subsequently followed by the addition of 500 *μ*L of acetic acid/HCl (pH 3.4) mixture and 500 *μ*L 0.8% thiobarbituric acid (TBA). This mixture was incubated at 100°C for 1 h. Thiobarbituric acid reactive species (TBARS) produced were measured at 532 nm in the JENWAY UV-visible spectrophotometer and the absorbance was compared with that of standard curve using malondialdehyde (MDA).

#### 2.2.6. Acetylcholinesterase (AChE) and Butyrylcholinesterase (BuChE) Inhibition Assay

The AChE inhibitory activity was determined according to the method of Ellman et al. [23] in a reaction mixture containing 200 *μ*L of AChE solution in 0.1 M phosphate buffer, pH 8.0, 100 *μ*L of a solution of 5,5-dithiobis-(2-nitrobenzoic) acid (3.3 mM DTNB in 0.1 M phosphate buffered solution, pH 7.0, containing 6 mM NaHCO_3_), 100 *μ*L of a solution of the inhibitor, and 500 *μ*L of phosphate buffer, pH 8.0. After incubation for 20 min at 25°C, 0.05 mM of acetylthiocholine iodide (100 *μ*L) was added as the substrate, and AChE activity was determined by UV-visible spectrophotometer from the absorbance changes at 412 nm for 3 min at 25°C.

100 *μ*L of 0.05 mM butyrylthiocholine iodide was used as a substrate to assay BuChE enzyme activity while all the other reagents and conditions were the same. The AChE and BuChE inhibitory activities were expressed as percentage inhibition.

#### 2.2.7. Data Analysis

The results of the three replicates were pooled and expressed as mean ± standard deviation (STD). A two-way analysis of variance (ANOVA) and the least significant difference (LSD) were also determined. Significance was accepted at *P* < 0.05.

## 3. Results

**Figure 1:**
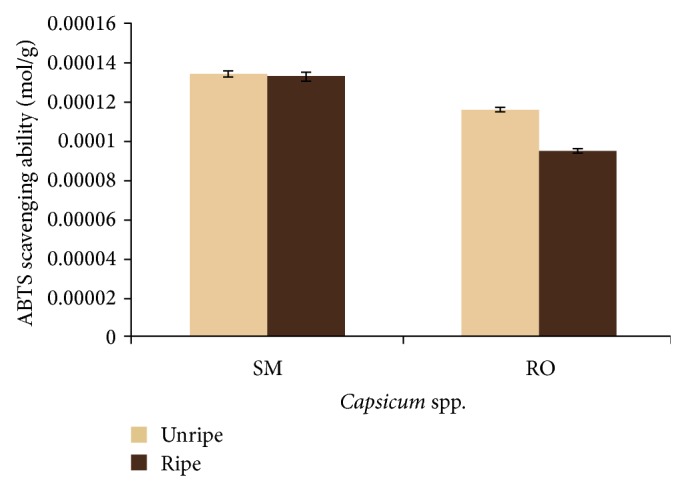
ABTS^*^ scavenging ability of* Capsicum *spp. SM:* Capsicum annuum* var.* accuminatum*. RO:* Capsicum chinense.*

**Figure 2:**
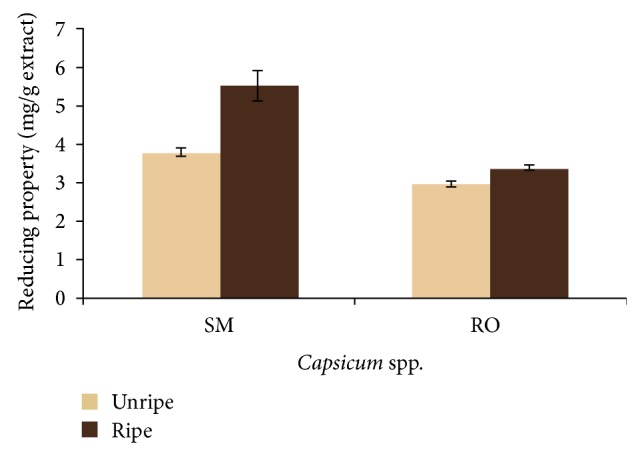
Ascorbic acid equivalent Ferric reducing property of aqueous extracts of* Capsicum *spp. SM:* Capsicum annuum* var.* accuminatum*. RO:* Capsicum chinense*.

The results for the total phenol content of the aqueous extract of ripe and unripe fruits of* Capsicum annuum *var.* accuminatum *(SM) and* Capsicum chinense* (RO) are presented in Table 1. There was no significant difference (*P* > 0.05) in the total phenol contents of the unripe (SM (14.09 mg/g); RO (14.17 mg/g)) and ripe (SM (13.28 mg/g); RO (13.66 mg/g))* Capsicum *spp. extracts. The ABTS^*^ scavenging ability reported as Trolox equivalent antioxidant capacity (TEAC) is presented in Figure 1. Extracts from both* Capsicum *spp. were able to scavenge ABTS^*^. There was no significant difference (*P* > 0.05) in the scavenging ability of the ripe and unripe SM samples, while unripe RO extracts showed a significantly higher (*P* < 0.05) scavenging ability than the ripe. The ferric reducing ability of the aqueous extracts reported as ascorbic acid equivalent as presented in Figure 2 revealed that the ripe SM and RO extracts had significantly higher (*P* < 0.05) ferric reducing properties than the unripe extracts of the two varieties.

**Figure 3:**
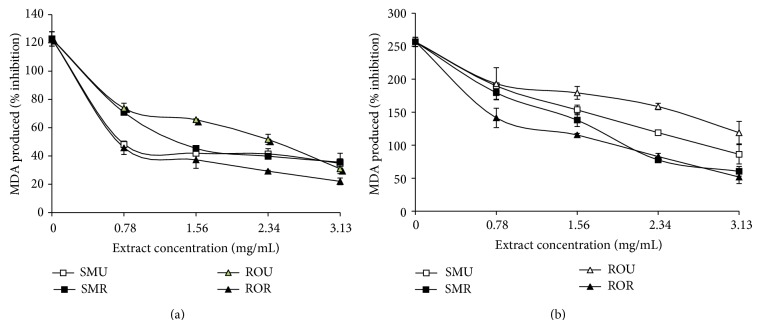
(a) Inhibition of Fe^2+^ induced lipid peroxidation in the brain by aqueous extract of* Capsicum *spp. (b) Inhibition of quinolinic acid induced lipid peroxidation in the brain by phenolic extracts of* Capsicum *spp. SMU:* Capsicum annuum* var.* accuminatum* (unripe). SMR:* Capsicum annuum* var.* accuminatum* (ripe). ROU:* Capsicum chinense* (unripe). ROR:* Capsicum chinense* (ripe).

**Figure 4:**
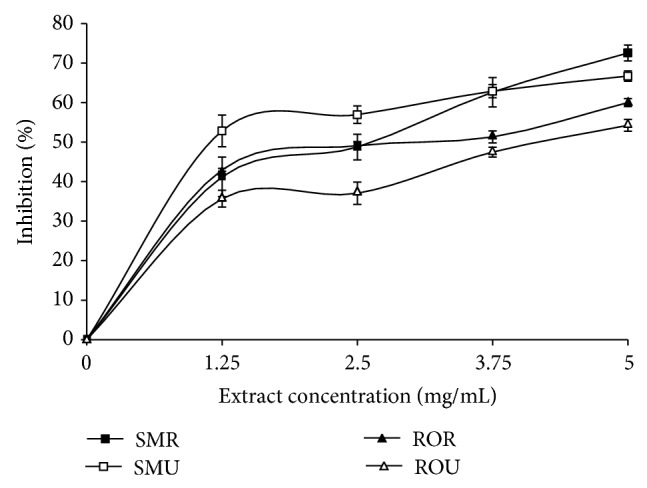
Inhibition of acetylcholinesterase by aqueous extract of* Capsicum *spp. SMU:* Capsicum annuum* var.* accuminatum* (unripe). SMR:* Capsicum annuum* var.* accuminatum* (ripe). ROU:* Capsicum chinense* (unripe). ROR:* Capsicum chinense* (ripe).

**Figure 5:**
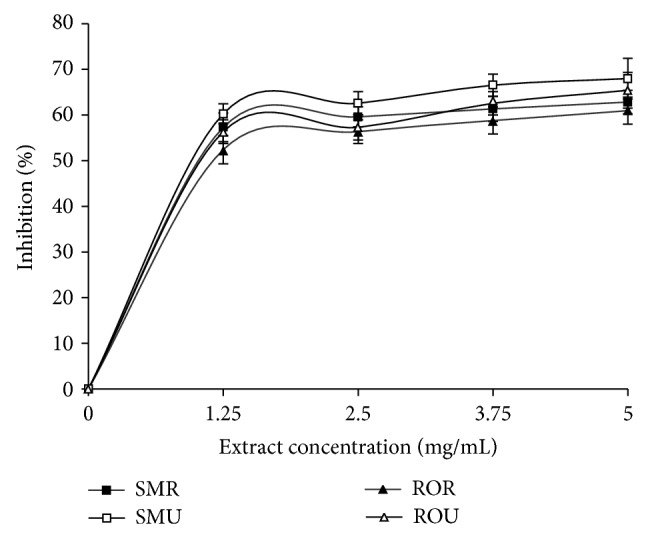
Butyrylcholinesterase inhibition by aqueous extract of* Capsicum *spp. SMU:* Capsicum annuum* var.* accuminatum* (unripe). SMR:* Capsicum annuum* var.* accuminatum* (ripe). ROU:* Capsicum chinense* (unripe). ROR:* Capsicum chinense* (ripe).

Incubation of rats' brain homogenate with 25 *μ*M Fe^2+^ resulted in a significant (*P* < 0.05) increase in brain MDA content (122.80%) as presented in Figure 3(a). However, the aqueous extracts of the two* Capsicum *spp. varieties were able to significantly (*P* < 0.05) lower the brain MDA content in a dose-dependent manner in the concentration range of 0.78 mg/mL–3.13 mg/mL. The unripe SM extracts (3.43 mg/mL) had higher inhibitory ability than the ripe extracts (3.63 mg/mL), while the ripe RO extracts (3.07 mg/mL) had higher inhibitory effect than the unripe (3.92 mg/mL) (Table 2). Furthermore, the ability of the aqueous extract from pepper fruits to inhibit quinolinic acid (QA) induced lipid peroxidation was assessed and presented in Figure 3(b). Incubation of rats brain homogenate with QA resulted in a significant (*P* < 0.05) increase in brain MDA content (256.67%). Aqueous extracts from the* Capsicum *spp. were also able to significantly (*P* < 0.05) lower the brain MDA content in dose-dependent manner. Table 2 revealed that the ripe samples (SM (4.87 mg/mL); RO (4.70 mg/mL)) had significantly higher inhibitory effect than the unripe samples (SM (5.70 mg/mL); RO (7.24 mg/mL)). The cholinesterase inhibitory activities of the pepper samples are presented in Figures 4 and 5 and Table 3. Ripe (3.27 mg/mL) and unripe (3.19 mg/mL) samples from SM had significantly higher AChE inhibitory abilities than RO (Ripe (3.72 mg/mL); unripe (4.27 mg/mL)) samples, while there was no significant difference in the BuChE inhibitory abilities of the unripe (SM (3.01 mg/mL); RO (3.19 mg/mL)) and ripe (SM (3.22 mg/mL); RO (3.38 mg/mL)).

## 4. Discussion

Oxidative damage caused by free radicals has been implicated in the development of neurodegenerative conditions [24, 25]. The brain is particularly susceptible to oxidative stress due to its high oxygen content [26]. Therefore, antioxidants could prove effective in the management of neurodegenerative conditions. Antioxidant capacity of the ripe and unripe pepper varieties was studied using a moderately stable nitrogen-centered radical species-ABTS radical [19 Re]. The ABTS^*^ scavenging properties of the extracts show their ability to prevent the initiation of oxidation chain reaction that ensues upon free radical generation [27]. Furthermore the extracts were also able to reduce Fe^3+^ to Fe^2+^ which is indicative of the reducing power of the extracts. The reversed reaction, which is the oxidation of Fe^2+^ to Fe^3+^, is a process by which iron can induce hydroxyl radicals generation in Fenton reaction [28]. The antioxidant properties of the pepper extracts could be linked to their phenolic content as the correlation between phenolic content and antioxidative properties has been established in studies involving a wide range of fruits and vegetables [29–31]. The antioxidant effects of phenolic compounds are attributed to the redox properties of their hydroxy groups achieved through several mechanisms including the scavenging of free radicals, chelation of transition metals serving as prooxidants, activation of antioxidant enzymes, and inhibition of oxidases [32, 33]. The higher phenolic content of the unripe* Capsicum *spp. under study may suggest that there could be a decline in the phenolics synthesis with the event of ripening.

Evidence for the contribution of lipid peroxidation in the brain to the development of neurodegenerative conditions has been found in postmortem brain tissue and patients suffering from Alzheimer's disease [26]. The high amount of polyunsaturated fatty acids and low antioxidant contents of the brain makes it susceptible to lipid peroxidation [34]. Lipid peroxidation is associated with loss of membrane fluidity and increase in permeability ultimately resulting in loss of membrane structure and function [35]. The increase in MDA content in the rat brain homogenates incubated with Fe^2+^ could be as result of the generation of hydroxy radicals generated from Fenton reaction. The decrease in brain MDA content by* Capsicum *spp. may be due to their ability to scavenge the hydroxyl radicals so generated. Quinolinic acid (QA) had been reported to activate neurons expressing NMDA receptors and glutamate-type excitotoxicity [36]. The mechanism through which QA induces lipid peroxidation has been linked to free radical generation resulting from overstimulation of NMDA receptors. Increases in QA concentration are known to be associated with several neurodegenerative diseases including Alzheimer's disease [37]. Free radical scavengers and antioxidant enzyme inducers can protect neuronal tissue against the oxidotoxicity of QA under* in vitro* and* in vivo* conditions [38, 39]. Thus the mechanism by which extracts from the* Capsicum *spp. were able to lower the increased generation of MDA in brain tissues could have been through scavenging of free radicals implicated in the overstimulation of NMDA receptors.

So far, inhibition of cholinesterases has proven to be the most effective approach in clinical management of AD. Acetylcholinesterase (AChE) and butyrylcholinesterase (BuChE) are the two cholinesterases present in the brain with acetylcholinesterase being the most dominant making up about 80% while the remaining 20% represents butyrylcholinesterase. Acetylcholine, a neuronal signaling molecule, is found to be depleted in AD brain and has been linked to decline in cognitive function observed in AD sufferers; hence acetylcholinesterase inhibitors (AChEI) have become important in the regulation of AChE and prolonging the acetylcholine levels at the synaptic clefts. The AChE inhibition by the pepper extracts used in this study agrees with previous studies where some plants and their products were found useful as acetylcholinesterase inhibitors (AChEI), some of which include* Ginkgo biloba* [40] and* Quisqualis indica* Linn [41]. Furthermore, inhibition of BuChE is also considered as a therapeutic strategy, in the AD brain, as it has been implicated in the aggregation of amyloid beta peptides associated with neurodegenerative processes and observed clinical dementia [42]. The ratio of BuChE to AChE changes drastically from about 0.5 to as high as 11. This increase in BuChE and decline in AChE suggest that BuChE could be a more important therapeutic target. It is therefore noteworthy that the extracts showed a higher inhibition for BuChE than AChE [43].

## 5. Conclusion

This study has shown that aqueous extract from* Capsicum *spp. have strong antioxidant capacity. The ripe fruits however displayed a more potent antioxidant capacity compared to their unripe counterparts. Furthermore, these extracts were also able to inhibit the two cholinesterases linked to AD. This is indicative of possible nutritional strength of* Capsicum *spp. as a possible dietary source of palliative/preventive targets for AD.

## Figures and Tables

**Table 1:** Total phenolic content of the aqueous extract of *Capsicum *spp. (mg/g sample).

	Unripe	Ripe
SM	14.09 ± 1.09^a^	13.28 ± 0.93^a^
RO	14.17 ± 1.27^a^	13.66 ± 1.09^a^

Values represent means of triplicate. Values with the same letter along the same column are not significantly different (*P* > 0.05). SM: *Capsicum annuum* var. *accuminatum*; RO: *Capsicum chinense*.

**Table 2:** EC_50_ values for inhibition of Fe^2+^ and quinolinic acid induced lipid peroxidation by *Capsicum *spp. (mg/mL).

Type of pepper	Fe^2+^ induced		QA induced	
	Unripe	Ripe	Unripe	Ripe
SM	3.43 ± 0.09^a^	3.63 ± 0.04^b^	5.70 ± 0.07^a^	4.87 ± 0.06^a^
RO	3.92 ± 0.11^a^	3.07 ± 0.09^a^	7.24 ± 0.09^b^	4.70 ± 0.04^a^

Values represent means of triplicates. Values with the same letter along the same column are not significantly different (*P* > 0.05). SM: *Capsicum annuum* var. *accuminatum;* RO: *Capsicum chinense*.

**Table 3:** EC_50_ values for inhibition of acetylcholinesterase and butyrylcholinesterase in mg/mL.

Type of pepper	AChE		BuChE	
	Unripe	Ripe	Unripe	Ripe
SM	3.19 ± 0.04^a^	3.27 ± 0.09^a^	3.01 ± 0.03^a^	3.22 ± 0.05^a^
RO	4.27 ± 0.03^b^	3.72 ± 0.04^b^	3.19 ± 0.04^a^	3.38 ± 0.02^a^

Values represent means of triplicate. Values along the same column with the same letter are not significantly different. AChE: acetylcholinesterase; BuChE: butyrylcholinesterase; SM: *Capsicum annuum* var. *accuminatum*; RO: *Capsicum chinense*.
